# A review of the epidemiology and treatment of orthopaedic injuries after earthquakes in developing countries

**DOI:** 10.1186/s13017-017-0115-8

**Published:** 2017-02-10

**Authors:** James S. MacKenzie, Bibek Banskota, Norachart Sirisreetreerux, Babar Shafiq, Erik A. Hasenboehler

**Affiliations:** 1 Department of Orthopaedic Surgery, The Johns Hopkins University, 601 N. Caroline Street, Baltimore, 21287 MD USA; 2 Department of Orthopaedics, Hospital and Rehabilitation Centre for Disabled Children, Adhikari Gaoun, Urgratara VDC-6, Janagal Kavre Nepal; 3 Department of Orthopaedics, Faculty of Medicine, Ramathibodi Hospital, Mahidol University, 270 Rama VI Rd., Ratchatewi, Bangkok, 10400 Thailand; 4 Department of Orthopaedic Surgery, The Johns Hopkins University/Johns Hopkins Bayview Medical Center, 4940 Eastern Ave., #A667, Baltimore, 21224-2780 MD USA

**Keywords:** Developing countries, Earthquake, Epidemiology, Orthopaedic injury

## Abstract

**Background:**

Earthquakes in developing countries are devastating events. Orthopaedic surgeons play a key role in treating earthquake-related injuries to the extremities. We describe orthopaedic injury epidemiology to help guide response planning for earthquake-related disasters.

**Methods:**

Several databases were searched for articles reporting primary injury after major earthquakes from 1970 to June 2016. We used the following key words: “earthquake” AND “fracture” AND “injury” AND “orthopedic” AND “treatment” AND “epidemiology.” The initial search returned 528 articles with 253 excluded duplicates. The remaining 275 articles were screened using inclusion criteria, of which the main one was the description of precise anatomic location of fracture. This yielded 17 articles from which we analyzed the ratio of orthopaedic to nonorthopaedic injuries; orthopaedic injury location, type, and frequency; fracture injury characteristics (open vs. closed, single vs. multiple, and simple vs. comminuted); and first-line treatments.

**Results:**

Most injuries requiring treatment after earthquakes (87%) were orthopaedic in nature. Nearly two-thirds of these injuries (65%) were fractures. The most common fracture locations were the tibia/fibula (27%), femur (17%), and foot/ankle (16%). Forty-two percent were multiple fractures, 22% were open, and 16% were comminuted. The most common treatment for orthopaedic injuries in the setting of earthquakes was debridement (33%).

**Conclusions:**

Orthopaedic surgeons play a critical role after earthquake disasters in the developing world. A strong understanding of orthopaedic injury epidemiology and treatment is critical to providing effective preparation and assistance in future earthquake disasters.

## Background

**Fig. 1:**
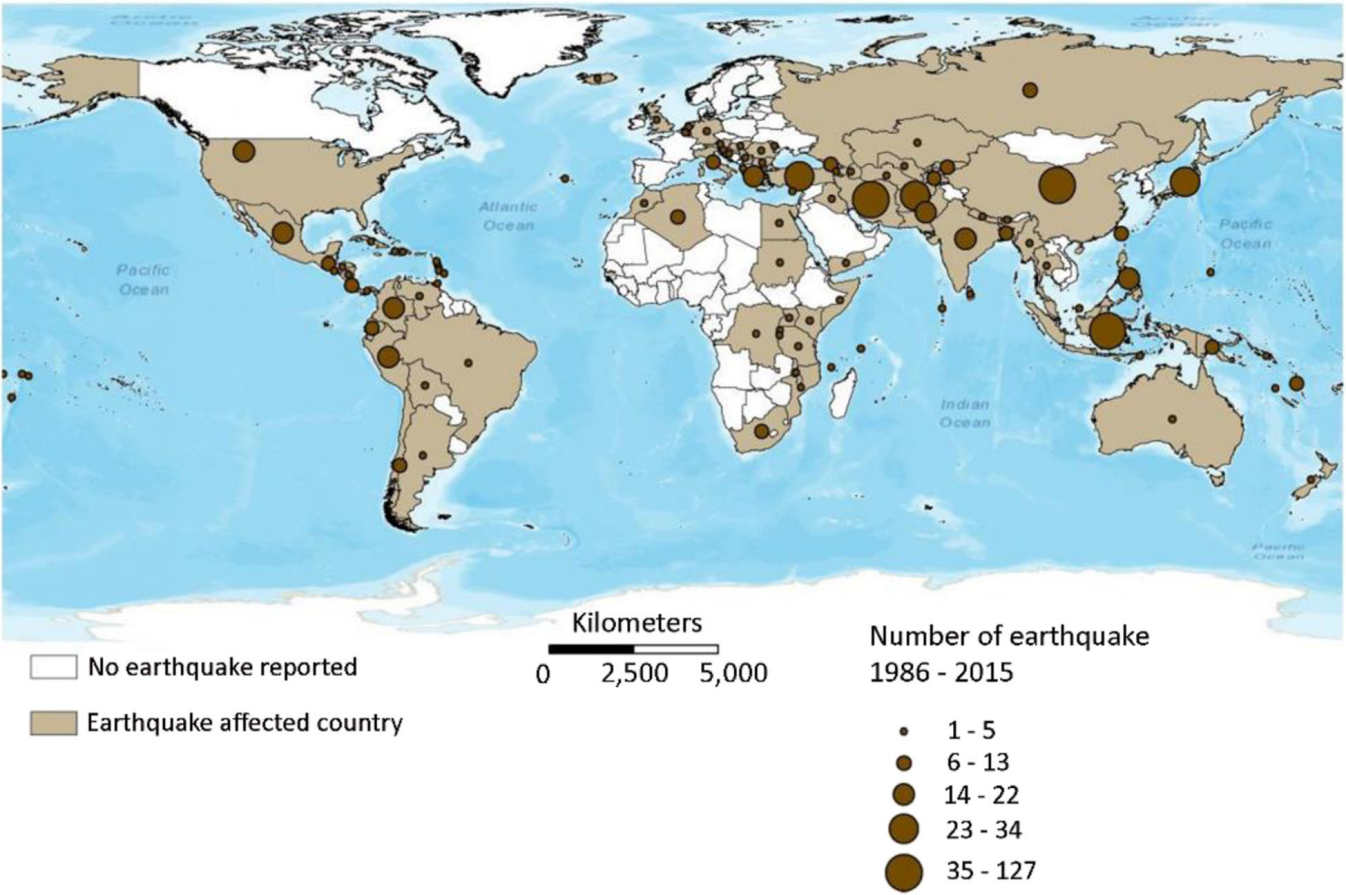
Number of occurrences of earthquake disasters by country, 1986–2015. Reprinted from D. Guha-Sapir - EM-DAT: The CRED/OFDA International Disaster Database – www.emdat.be – Université Catholique de Louvain – Brussels – Belgium

Since 2000, major earthquakes have taken more than 800,000 lives and injured countless more [1]. As defined by the Centre for Research on the Epidemiology of Disasters, the mean number of “major” earthquakes annually (defined as causing more than 10 deaths, affecting more than 100 people, and resulting in international aid or declaration of a state of emergency) was 21 from 1970 to 2005 [2, 3]. From 2000 to 2005, that mean increased to more than 30 because of increasing population density in seismically active regions [2]. Figure 1 shows the number of earthquake disasters by country from 1974 to 2003 [3]. As population density continues to increase in these areas, challenges grow for emergency responders in the aftermath of major earthquakes.

After an earthquake, the local medical infrastructure is often damaged or destroyed [4, 5]. International responders may not be able to rely on the medical resources of the afflicted region. Therefore, humanitarian organizations must provide the necessary medical equipment and supplies. This requires planning, efficient resource allocation, and an understanding of the types of injuries that are likely to be encountered.

Many survivable injuries are orthopaedic in nature; thus, orthopaedic surgeons play a key role in providing care to earthquake survivors. Long bone fractures, major soft tissue injuries, and crush injuries to the extremities are typically survivable with proper treatment [5]. Understanding the epidemiology and treatment of orthopaedic injuries after an earthquake is paramount to planning an effective response.

Although primary data on the anatomic locations of orthopaedic extremity injuries exist for individual major earthquakes, no review has compiled detailed epidemiologic data from these studies. Several reviews have more broadly characterized injury patterns as “lower limb” or “upper limb” and “open” or “closed” [5, 6] but do not contain more detailed epidemiologic information.

The purpose of this review was to compile specific epidemiologic and treatment information on orthopaedic extremity injuries after major earthquakes, including the ratio of orthopaedic to nonorthopaedic injuries; orthopaedic injury location, type, and frequency; fracture injury characteristics (open vs. closed, single vs. multiple, and simple vs. comminuted); and first-line treatments. We hope to provide orthopaedic surgeons and humanitarian aid organizations with more precise information on the types of extremity injuries they are likely to encounter to help them more confidently estimate what surgical supplies and equipment are necessary for their treatment.

## Methods

We searched the PubMed/MEDLINE, Cochrane Library, National Library of Medicine: Disaster Lit, WHO Global Index Medicus, and EMbase databases from 1970 to June 2016 for articles reporting primary injury data from major earthquakes using the following key words: “earthquake” AND “fracture” AND “injury” AND “orthopedic” AND “treatment” AND “epidemiology.” The initial search returned 528 articles with 253 duplicates, which were excluded. The remaining 275 articles were screened using inclusion criteria established prior to the search. The primary criterion for inclusion was a description of injury epidemiology by fracture location in a minimum of 8 categories: ankle/foot, clavicle/scapula, femur, humerus, pelvis, radius/ulna, tibia/fibula, and wrist/hand. Articles that classified injuries simply as occurring in the “upper limb” vs. “lower limb” or as “open” vs. “closed” were excluded, as were articles written in languages other than English. This resulted in 17 articles.

From these articles, we extracted data on the number of orthopaedic vs. nonorthopaedic injuries, orthopaedic injury types, fracture characteristics, and treatments provided. The primary criterion for article selection in analysis of orthopaedic to nonorthopaedic injury was inclusion of data on nonorthopaedic injuries in the following regions: head, thoracic, and abdominal. Articles that did not include data on nonorthopaedic injuries were excluded from this subanalysis. To facilitate analysis across studies for location of fracture injury, we grouped certain anatomical locations together (i.e., ankle/foot, clavicle/scapula, radius/ulna, tibia/fibula, and wrist/hand) even when certain articles had provided more granular detail. The level of detail describing the type of treatment varied across studies. For studies that noted closed reduction/internal fixation (CRIF) and closed reduction separately, we combined these categories. Criteria for inclusion in analysis of type of orthopaedic injury were designed to preserve accuracy of the relative proportions of each injury type. Studies had to present data on the same 5 types of orthopaedic injury: compartment syndrome, crush injury, crush syndrome, fracture, and major soft tissue injury. Only major soft tissue injury was considered in this analysis. Studies that did not differentiate major soft tissue injury from minor soft tissue injury were excluded.

## Results

### Orthopaedic vs. nonorthopaedic injuries

Six of the 17 articles contained data on nonorthopaedic injuries, as well as orthopaedic injuries [7–13]. The overwhelming majority of survivable injuries sustained in earthquakes are orthopaedic in nature (Table 1). These 6 articles reported 1549 earthquake-related injuries. Of these, 87% were orthopaedic and 13% were nonorthopaedic (5.4% head, 4.4% thoracic, and 3.4% abdominal).

**Table 1:** Classification of type of 1549 injuries sustained in earthquakes

Study	No. of injuries reported			
	Orthopaedic	Nonorthopaedic		
		Head	Thoracic	Abdominal
Tahmasebi et al. [13]	228	32	17	18
Gormeli et al. [8]	260	14	17	17
Dai et al. [7]	258	8	15	9
Kaim Khani et al. [9]	150	8	6	3
Roy et al. [11]	125	9	3	3
Phalkey et al. [10]	324	12	11	2
Total	1345 (87%)	83 (5.4%)	69 (4.4%)	52 (3.4%)

### Anatomic location of fracture

In regard to anatomic location of fracture, all 17 articles reporting on 8 major earthquakes were used because this was the primary inclusion criterion for the review. These articles reported 3988 fractures (Table 2). No articles prior to the 1972 Nicaragua earthquake contained data on precise anatomic location of injury. Because most studies did not record frequency of patellar fracture, this category was omitted from the combined data. We found that 2372 of 3988 fractures (59%) involved the lower extremity. The distribution of fractures by category was as follows: 27%, tibia/fibula; 17%, femur; 16% ankle/foot; 12%, radius/ulna; 9.6% pelvis; 8.4%, humerus; 6.9% wrist/hand; and 3.3%, clavicle/scapula. Together, 44% of fractures involved the tibia/fibula or the femur.

**Table 2:** Anatomic location of 3988 fractures sustained in earthquakes

Study by country (year) of earthquake	Tibia/fibula	Femur	Ankle/foot^a^	Radius/ulna	Pelvis^b^	Humerus	Wrist/hand^c^	Clavicle/scapula
Nepal (2015)								
Vaiysa [24]	14	17	2	16	NR	9	2	NR
Bar-On [16]	34	10	30	11	9	7	7	4
Turkey (2011)								
Guner [15]	100	36	64	70	61	23	23	22
Gormeli [8]	77	12	42	37	51	17	13	18
Haiti (2010)								
Bar-On [14]	109	95	23	26	38	36	18	4
Blumberg [25]	41	66	53	14	34	18	24	3
China (2008)								
Dai [7]	81	38	48	30	29	23	10	15
Chen [17]	271	140	170	138	NR	70	69	NR
Xiang [26]	19	23	16	13	12	12	NR	1
Pakistan (2005)								
Kaim Khani [9]	45	23	24	14	8	8	15	3
Rajpura [27]	24	11	11	9	2	7	7	NR
Bozkurt [18]	45	42	34	20	45	26	45	10
Iran (2003)								
Emami [4]	29	21	2	15	17	22	5	9
Tahmasebi [13]	25	28	12	15	17	6	6	6
India (2001)								
Roy [11]	20	17	19	7	15	8	NR	NR
Phalkey [10]	113	65	26	28	20	21	3	5
Nicaragua (1972)								
Whittaker [28]	27	21	57	30	24	21	30	30
Total	1074 (27%)	665 (17%)	633 (16%)	493 (12%)	382 (9.6%)	334 (8.4%)	277 (6.9%)	130 (3.3%)

*Abbreviations*: *NR* not reported

^a^Includes fractures of the tarsus, metatarsus, and phalanges

^b^Includes fractures of the acetabulum

^c^Includes fractures of the carpus, metacarpus, and phalanges

### Treatment types

In regard to treatments, 5 articles provided data that could be aggregated. From these 5 articles, 1260 procedures were reported with the following frequencies: 33%, debridement; 24%, closed reduction/casting/CRIF; 24%, open reduction and internal fixation (ORIF); 12%, external fixation; and 7.5%, amputation (Table 3). The relative frequency of different treatment types varied across studies. Bar-On et al. [14] reported the use of external fixation in 31% of cases, whereas Phalkey et al. [10] reported it in fewer than 2% of cases.

**Table 3:** Type of initial treatment of 1260 orthopaedic injuries sustained in earthquakes, by study

Study	Debridement	CR/casting/CRIF	ORIF	External fixation	Amputation
Vaiysa et al. [24]	24	11	43	9	15
Guner et al. [15]	166	138	117	37	12
Gormeli et al. [8]	45	29	30	22	7
Bar-On et al. [14]	91	27	18	73	23
Phalkey et al. [10]	87	100	93	5	38
Total	413 (33%)	305 (24%)	301 (24%)	146 (12%)	95 (7.5%)

*Abbreviations*: *CR* closed reduction, *CRIF* closed reduction/internal fixation, *ORIF* open reduction/internal fixation

### Injury types

A breakdown of the types of orthopaedic injuries was extracted from 4 studies of 3 earthquakes [7, 8, 13, 15]. A total of 1365 orthopedic injuries were included in our analysis (Table 4). Fracture was the most common orthopaedic injury, accounting for approximately 65%. Crush injury accounted for 13% of injuries, followed by compartment syndrome (7.6%), major soft-tissue injury (7.3%), and crush syndrome (6.4%).

**Table 4:** Types of orthopaedic injuries (*n* = 1365) sustained in earthquakes, by study

Study	No. of injuries				
	Fracture	Crush injury	Compartment syndrome	Major soft tissue	Crush syndrome
Tahmasebi et al. [13]	147	22	18	35	6
Guner et al. [15]	442	72	40	24	41
Gormeli et al. [8]	144	46	28	20	22
Dai et al. [7]	160	40	18	21	19
Total	893 (65%)	180 (13%)	104 (7.6%)	100 (7.3%)	88 (6.4%)

### Fracture characteristics

Four studies reporting 1363 fractures differentiated between single (58%) vs. multiple (42%) fracture (Table 5). Three studies reporting 746 fractures distinguished between simple (84%) and comminuted (16%) fractures. Six studies reporting 1372 fractures distinguished between closed (78%) and open (22%) fractures.

**Table 5:** Characteristics of fractures sustained in earthquakes

Study	No. of fractures					
	Multiple fracture		Comminuted fracture		Open fracture	
	No	Yes	No	Yes	No	Yes
Bar-On et al. [14]	NR	NR	NR	NR	261	99
Bar-On et al. [16]	NR	NR	NR	NR	89	37
Chen et al. [17]	287	336	NR	NR	NR	NR
Dai et al. [7]	42	112	109	51	105	55
Gormeli et al. [8]	70	74	97	47	123	21
Guner et al. [15]	386	56	423	19	405	37
Kaim Khan et al. [9]	NR	NR	NR	NR	92	48
Total	785 (58%)	578 (42%)	629 (84%)	117 (16%)	1075 (78%)	297 (22%)

*Abbreviations*: *NR* not reported

## Discussion

The type and number of injuries caused by an earthquake vary according to human/individual factors, seismic/geologic factors, and the built environment [2]. Despite this variation, patterns in injury can be observed across different earthquakes. Such trends include the relative frequency, type, location, and treatment of orthopaedic injuries. The vast majority of injuries treated after earthquakes are orthopaedic in nature [5–12, 15]. Nonorthopaedic injuries to the head, chest, and abdomen account for 13% of injuries after earthquakes and are usually considered to be unsurvivable [5]. Therefore, patients who present to healthcare facilities are more likely to have extremity injuries than nonorthopaedic injuries [5].

Most orthopaedic injuries are fractures, and after an earthquake, most fractures are in the diaphyseal region of the femur and tibia [2]. This, coupled with the high frequency of pelvic and humeral fractures, indicates that large-bone fractures are more common after earthquake than injury to the smaller bones of the forearm, wrist, ankle, foot, and hand (62% vs. 35%). This necessitates that approximately twice as many femoral, pelvic, and tibial orthopaedic implants and instruments be brought to earthquake disaster zones compared with supplies specific to upper-extremity fractures. In particular, tibial instruments are most needed because this is typically the most common location of fracture following earthquakes [4, 7–11, 14–17]. Bozkurt et al. [18] noted that most tibial fractures (84%) after the 2005 Pakistan earthquake involved the middle and distal tibial shaft as opposed to the proximal tibia or tibial plateau.

Characterizations of fracture as single vs. multiple, open vs. closed, and simple vs. comminuted are important considerations when treating the earthquake victim with fracture injury because initial treatment and fixation technique is influenced by these characteristics and, therefore, so is response planning. High-energy crush injuries from falling debris may cause long-bone fractures with a high degree of comminution. Comminuted fractures are much more likely to occur as a result of earthquakes vs. other causes [19]. We found that 16% of fractures after earthquakes were comminuted. Open and multiple fractures are also common after earthquakes. According to our review, 22% of fractures were open and 42% of cases involved multiple fractures.

One might expect the proportions of crush injuries, comminuted fractures, and open fractures to be higher in the setting of earthquake disasters. No reviewed studies commented on these numbers; however, we speculate that they likely reflect a multifactorial process. On the basis of the experience of the 2015 Nepal earthquake, injury patterns vary according to factors such as the location of the person when the injury is sustained (i.e., indoors vs. outside), the type and amount of building construction material used (e.g., relatively small village domiciles vs. large urban structures with heavy construction materials), and the mechanism of injury (e.g., falling debris vs. falling from height).

In the immediate aftermath of a massive earthquake, it is often unrealistic to pursue definitive internal fixation, and damage-control orthopedics (DCO) may be the approach of choice until definitive fixation is possible. The focus should be on hemorrhage management, wound debridement, infection control, and soft tissue stabilization. External fixation is key to proper management of fractures and soft tissue stabilization, yielding favorable results in earthquake disaster scenarios [20–22]. The ratio of external fixation to ORIF depends largely on when the response team arrives at the earthquake location [22]. For example, in the Haiti earthquake of 2010, the Israeli Defense Force reported that it took approximately 2 weeks for an adequate number of treatment centers to be established to allow definitive fixation [22]. They noted that the use of external fixation and, when necessary, amputation, as a means of DCO allowed fractures to be definitively addressed later by more sufficiently staffed and supplied treatment teams or allowed patients to be transported to better-equipped facilities [22]. Awais et al. noted similar findings, advocating for the use of both external fixation and, when necessary, amputation, after the 2005 earthquake in Pakistan, which claimed more than 73,000 lives [20, 23]. A retrospective study after that earthquake reported that in 295 of 1145 fractures (26%), reductions were achieved with external fixation [20]. We did not expect to find a discrepancy between open fracture rates (22%) and the rate of surgical debridement (33%). As irrigation and debridement is key for the management of open fractures, an explanation for this difference might be that grossly contaminated open fracture wounds may require multiple debridement before definitive fixation and wound closure can be achieved.

Compared with internal fixation, external fixation reduces the risk of operative infection, minimizes operative time, and is technically easier to perform when intraoperative imaging is unavailable [20]. Although we found that only 12% of fractures were stabilized by external fixation, the use of external fixation varied by study from less than 2% to more than 30% [10, 14]. Response teams arriving early to earthquake sites should bring a high proportion of external fixators and be prepared to reduce as many as 25% of fractures by external fixation; several retrospective reviews have proven the technique’s value after disaster [8, 14, 20, 22].

This review is limited by the level of detail provided by the included studies. Few studies precisely describe the fracture fixation methods used and the exact anatomic location of fractures. Future epidemiological studies, in addition to characterizing specific injury location, should focus on how much and what kind of surgical supplies were needed for each operative fixation. Another weakness of this study is that although we were able to aggregate and concisely present a large number of studies related to orthopaedic earthquake injury, the review did not follow the PRISMA or similar methodology for systematic reviews and thus cannot be considered as such. The strengths of the study include the level of detail in describing orthopaedic injury epidemiology after earthquakes, the large amount of data included, and the fact that it is the first study to aggregate such information for use by orthopaedic surgeons who travel to earthquake disaster zones.

## Conclusion

As population density increases in the developing world, the chance of mass casualties caused by earthquakes will increase. It is vital that first responders, including orthopaedic surgeons, be equipped to handle the challenging environments and injuries they will encounter. To accomplish that, a thorough understanding of the nature and epidemiology of the injuries is paramount and will enable response teams around the world to better serve those injured by earthquakes.

## Abbreviations

CRIF: Closed reduction/internal fixation
DCO: Damage-control orthopedics
ORIF: Open reduction/internal fixation
